# Comparison between the comet assay and pimonidazole binding for measuring tumour hypoxia

**DOI:** 10.1054/bjoc.2000.1489

**Published:** 2000-11-22

**Authors:** P L Olive, R E Durand, J A Raleigh, C Luo, C Aquino-Parsons

**Affiliations:** B.C. Cancer Research Centre/B.C. Cancer Agency, Vancouver, V5Z 1L3, Canada; University of North Carolina School of Medicine, Chapel Hill, NC, 27599–7512, USA

**Keywords:** tumour hypoxia, pimonidazole, comet assay

## Abstract

Pimonidazole is finding increasing use in histochemical analyses of hypoxia in tumours. Whether it can identify every hypoxic cell in a tumour, and whether the usual subjective criteria used to define ‘positive’ cells are optimal, are less certain. Therefore, our aim was to develop an objective flow cytometry procedure for quantifying pimonidazole binding in tumours, and to validate this method by using a more direct indicator of radiobiologic hypoxia, the comet assay. SCCVII tumours in C3H mice were analysed for pimonidazole binding using flow cytometry and an iterative curve-fitting procedure, and the results were compared to the comet assay for the same cell suspensions. On average, cells defined as anoxic by flow analysis (n = 43 tumours) bound 10.8 ± 0.95 times more antibody than aerobic cells. In samples containing known mixtures of aerobic and anoxic cells, hypoxic fractions as low as 0.5% could easily be detected. To assess the flow cytometry assay under a wider range of tumour oxygen contents, mice were injected with hydralazine to reduce tumour blood flow, or allowed to breathe various gas mixtures during the 90 min exposure to pimonidazole. Hypoxic fraction estimated by the pimonidazole binding method agreed well with the hypoxic fraction measured using the comet assay in SCCVII tumours (r2 = 0.87, slope = 0.98), with similar results in human U87 glioma cells and SiHa cervical carcinoma xenografts. We therefore conclude that this objective analysis of pimonidazole labelling by flow cytometry gives a convenient and accurate estimate of radiobiological hypoxia. Preliminary analyses of biopsies from 3 patients given 0.5 g m–2 pimonidazole also suggest the suitability of this approach for human tumours. © 2000 Cancer Research Campaign http://www.bjcancer.com


*British Journal of Cancer* (2000) **83**(11), 1525–1531
© 2000 Cancer Research Campaign
doi: 10.1054/ bjoc.2000.1489, available online at http://www.idealibrary.com on IDEAL®

http://www.bjcancer.com

### Comparison between the comet assay and pimonidazole binding for measuring tumour hypoxia

**PL Olive[1], RE Durand[1], JA Raleigh[2], C Luo[1] and C Aquino-Parsons[1]**

[1]B.C. Cancer Research Centre/B.C. Cancer Agency, Vancouver, Canada, V5Z 1L3 and [2]University of North Carolina School of Medicine, Chapel Hill, NC, 27599–7512, USA

Summary Pimonidazole is finding increasing use in histochemical analyses of hypoxia in tumours. Whether it can identify every hypoxic cell in a tumour, and whether the usual subjective criteria used to define 'positive' cells are optimal, are less certain. Therefore, our aim was to develop an objective flow cytometry procedure for quantifying pimonidazole binding in tumours, and to validate this method by using a more direct indicator of radiobiologic hypoxia, the comet assay. SCCVII tumours in C3H mice were analysed for pimonidazole binding using flow cytometry and an iterative curve-fitting procedure, and the results were compared to the comet assay for the same cell suspensions. On average, cells defined as anoxic by flow analysis (n = 43 tumours) bound 10.8 ± 0.95 times more antibody than aerobic cells. In samples containing known mixtures of aerobic and anoxic cells, hypoxic fractions as low as 0.5% could easily be detected. To assess the flow cytometry assay under a wider range of tumour oxygen contents, mice were injected with hydralazine to reduce tumour blood flow, or allowed to breathe various gas mixtures during the 90 min exposure to pimonidazole. Hypoxic fraction estimated by the pimonidazole binding method agreed well with the hypoxic fraction measured using the comet assay in SCCVII tumours (r2 = 0.87, slope = 0.98), with similar results in human U87 glioma cells and SiHa cervical carcinoma xenografts. We therefore conclude that this objective analysis of pimonidazole labelling by flow cytometry gives a convenient and accurate estimate of radiobiological hypoxia. Preliminary analyses of biopsies from 3 patients given 0.5 g m–2 pimonidazole also suggest the suitability of this approach for human tumours. © 2000 Cancer Research Campaign http://www.bjcancer.com

**Keywords:** tumour hypoxia; pimonidazole; comet assay

Hypoxic cells present in many solid tumours can limit tumour cure by ionizing radiation and perhaps by some drugs (Bush et al, 1978; Overgaard, 1994; Hockel et al, 1996; Fyles et al, 1998). Detecting hypoxic tumour cells at the start of therapy would provide an opportunity to include treatment strategies designed to improve tumour oxygenation or kill hypoxic tumour cells (Brown and Giaccia, 1998). Pimonidazole is known to be preferentially bound by hypoxic tumour cells, so detection of pimonidazole adducts using monoclonal antibodies can serve as a method for measuring tumour hypoxia (Thrall et al, 1997; Raleigh et al, 1999). Clinical trials of pimonidazole binding in human tumours are currently underway in the US, Canada and Europe, with the goal of determining whether this marker can be used to predict tumour response to radiation (Kennedy et al, 1997; Varia et al, 1998). Although most efforts have been directed toward analysis of antibody binding in tumour sections, flow cytometry should also be applicable. Flow cytometry offers certain advantages over static cytometry in terms of speed of analysis, removal of necrotic cells, and potential for multi-parameter analyses. However, the exact percentage of radiobiologically hypoxic cells can be difficult to estimate from flow histograms because hypoxia marker binding in single cells is typically seen as a continuum (Lee et al, 1996; Durand and Raleigh, 1998; Kavanagh et al, 1999).

*Received 3 April 2000*
*Revised 24 July 2000*
*Accepted 1 August 2000*

*Correspondence to:* PL Olive

Further complications arise from the fact that different tumours metabolize and bind pimonidazole at different rates, and in the clinical situation, it is difficult to determine the optimal dose for best visualizing the hypoxic cells of a particular tumour. This was recognized in a previous study where the number of cells with pimonidazole adducts exceeding an arbitrary threshold was found to correlate exactly with cellular radiosensitivity only when labelling was optimized (Durand and Raleigh, 1998). However, it was also noted in that study that those tumours with higher levels of hypoxia could be identified even with non-optimal labelling concentrations of pimonidazole, which in turn suggests that a more rigorous and objective analysis of the flow histograms might be useful.

Since the ultimate variable of interest is the number of radiobiologically hypoxic cells, and since the alkaline comet assay can detect those cells (Olive and Durand, 1992; Olive et al, 1997) even in the clinic (Olive et al, 1993; McLaren et al, 1997; Aquino-Parsons et al, 1999), we postulated that the comet assay could be used to 'calibrate' the analysis of pimonidazole histograms obtained with flow cytometry. A unique advantage of the comet assay is that the relation between oxygen concentration and radiation-induced DNA single-strand breaks is known to be identical to the relation between oxygen concentration and radiation-induced cell killing (Chapman et al, 1974; Zhang et al, 1995). Since both the comet assay and pimonidazole binding can easily be assessed in any population of single cells, it is possible to directly compare the two methods. A murine tumour and two human tumour xenografts were therefore examined using both comet and pimonidazole marker binding under a range of tumour oxygenations produced by breathing different gas mixtures. From these data, we developed an iterative curve-fitting procedure to objectively estimate the radiobiologically hypoxic fraction of cells from the pimonidazole binding distributions. The fitting methodology also appeared practical for biopsies in an initial series from cervical cancer patients who had received pimonidazole.

### MATERIALS AND METHODS

### Cells and tumours

Chinese hamster V79-171b cells were maintained in exponential growth by subcultivating twice weekly in minimal essential medium containing 10% fetal bovine serum. SCCVII squamous cell carcinoma cells were transplanted subcutaneously over the sacral region of inbred male C3H/HeN mice, approximately 30 g in weight. U87 glioma cells and SiHa cervical carcinoma cells were implanted subcutaneously in the back of NOD/SCID mice ($10^6$ cells/mouse in 0.1 ml phosphate buffered saline (PBS)). Tumours were used for experimentation 2–4 weeks later when they had reached a weight of approximately 400 mg.

Mice were injected intraperitoneally with 100 mg $kg^{-1}$ pimonidazole (Hypoxyprobe-1, Natural Pharmacia International, Inc., Belmont, MA) from a stock solution of 20 mg $ml^{-1}$ in sterile PBS. For 5 minutes before and until time of sacrifice, mice breathed various humidified gas mixtures in a chamber supplied at a flow rate of 1.5 l $min^{-1}$: 100% $O_2$, carbogen (95% $O_2$, 5% $CO_2$), 452 ppm CO, or 10% $O_2$ in $N_2$. Alternatively, mice were injected with 5 mg $kg^{-1}$ hydralazine intravenously 15 min after administration of pimonidazole, or tumours were clamped 20 min after i.p injection of pimonidazole. 90 min after intraperitoneal injection of pimonidazole, corresponding to 3 plasma half-lives for this drug (Walton et al, 1989), mice were exposed to 12 Gy of 250 kV X-rays, immediately sacrificed, and the tumours excised within 1 min and placed in ice-cold PBS. A single cell suspension was prepared from the entire tumour by mincing in ice-cold PBS and filtering through 30 μm nylon mesh. Part of this suspension was used for the comet assay, and the remainder was incubated for 30 min at 37˚C with a mixture of trypsin, collagenase and DNAse as described previously (Olive, 1989). Cells were then filtered through 30 μm nylon mesh, centrifuged and resuspended in cold PBS. Cells were fixed in 70% ethanol and refrigerated overnight or up to several days before analysis of pimonidazole binding.

### Hoechst 33342 sorting of tumour cells

To examine pimonidazole binding in tumour cells as a function of their position relative to the functional vasculature, mice were injected intravenously with Hoechst 33342 20 min before sacrifice. Single cells from these tumours were then sorted on the basis of the Hoechst concentration gradient, as previously described (Olive, 1995).

### Analysis of pimonidazole binding in cervical tumours

Three patients with histologically confirmed locally advanced cervical cancer consented to undergo an excision biopsy after pimonidazole administration. For examination of tumour hypoxia, 1.0 g of Hypoxyprobe-1 was dissolved in 100 ml of 0.9% sterile saline at room temperature, and 0.5 g $m^{-2}$ was delivered as an intravenous infusion over about 20 min. Approximately 24 h later (about 4 plasma half-lives), a small excision biopsy was obtained. Biopsies were minced and incubated with trypsin, collagenase and DNAse for 30 minutes to provide a single cell suspension. Cells were filtered through 30 μm pore nylon mesh, suspended in PBS, and diluted with 95% ethanol to a 70% solution. Fixed cells were analysed for pimonidazole binding (generally within 2 weeks) as described below. Delayed analysis (up to 2 weeks) was not found to influence the percentage of hypoxic cells measured using flow cytometry.

### Flow cytometry analysis of pimonidazole binding

Ethanol-fixed cells were rinsed in phosphate buffer then resuspended in PST (PBS containing 4% serum and 0.1% triton X- 100). A fluorescein isothiocyanate (FITC)-conjugated (1:1000 dilution) or unconjugated (1:100 dilution) primary antibody were incubated with $2 \times 10^6$ alcohol-fixed cells for 2 h at 37˚C (for antibody source and description, see Raleigh et al (1999)). Samples were rinsed in PST and those cells that were incubated with the unconjugated primary were resuspended in a FITC conjugated secondary antibody diluted 1:100 in PST for 1 h at 37˚C. Samples were rinsed in PST and resuspended for DNA staining in 1 ml PBS containing 1 μg $ml^{-1}$ 4,6-diamidino-2-phenylindole dihydrochloride hydrate (DAPI). Samples were analysed on a Coulter Epics Elite cell sorter (Coulter Corp. Hialeah, FL). Approximately 20 000 cells were acquired for the mouse tumour studies, and 100 000 cells for cervical tumour analyses.

Univariate histograms, plotted as cell number versus logarithm of fluorescent antipimonidazole antibody intensity, were analysed by a least-squares approach for 3 Gaussian distributions representing aerobic, intermediate, and hypoxic tumour cell populations. No constraints on the positions of the distribution means were imposed. Therefore, the range but not absolute fluorescence intensity determined whether a hypoxic fraction could be reliably identified within cells from different tumours.

### Analysis of hypoxic fraction using the comet assay

Single cells (20 000) from irradiated tumour cell suspensions were embedded in 0.75% low gelling temperature agarose containing 2% dimethylsulphoxide and spread on agarose precoated microscope slides. Slides were carefully submersed in an alkaline, high salt lysing solution as previously described (McLaren et al, 1997). Following lysis and rinse in alkali, slides were electrophoresed in alkali at 0.6 volts $cm^{-1}$ for 25 min, and then stained for 20 min in 2.5 μg $ml^{-1}$ propidium iodide. For each sample, approximately 400 individual cells or 'comets' were observed using a Zeiss epifluorescence microscope and image analysis system (Olive et al, 1990). DNA damage was quantified as an increase in tail moment, an indicator of damage that is proportional to the number of strand breaks per cell. Tail moment was defined as the product of the percent of DNA (fluorescence) in the tail and the distance between the means of the head and tail fluorescence distributions.

For comet analysis of hypoxic fraction, frequency histograms for tail moment were used to calculate hypoxic fraction. An iterative fitting technique, independent of the amount of DNA damage, was used to determine the size and position of the two normal distributions representing the aerobic and hypoxic populations. The displacement between the aerobic and hypoxic peaks was constrained to fall between 1.9 and 3, and a free fit to the data was performed using a least squares approach (Olive et al, 1997).

*© 2000 Cancer Research Campaign*

### RESULTS

SCCVII tumour cells from air-breathing mice were analysed for pimonidazole binding with a FITC-conjugated monoclonal antibody using a bivariate presentation of DNA content versus log pimonidazole content (Figure 1). The diploid normal cell population is clearly visible in this tetraploid mouse tumour. These normal cells were primarily resident macrophages, with a hypoxic fraction similar to that of the tumour cells (Olive, 1989). The analysis of pimonidazole binding in the SCCVII tumour was

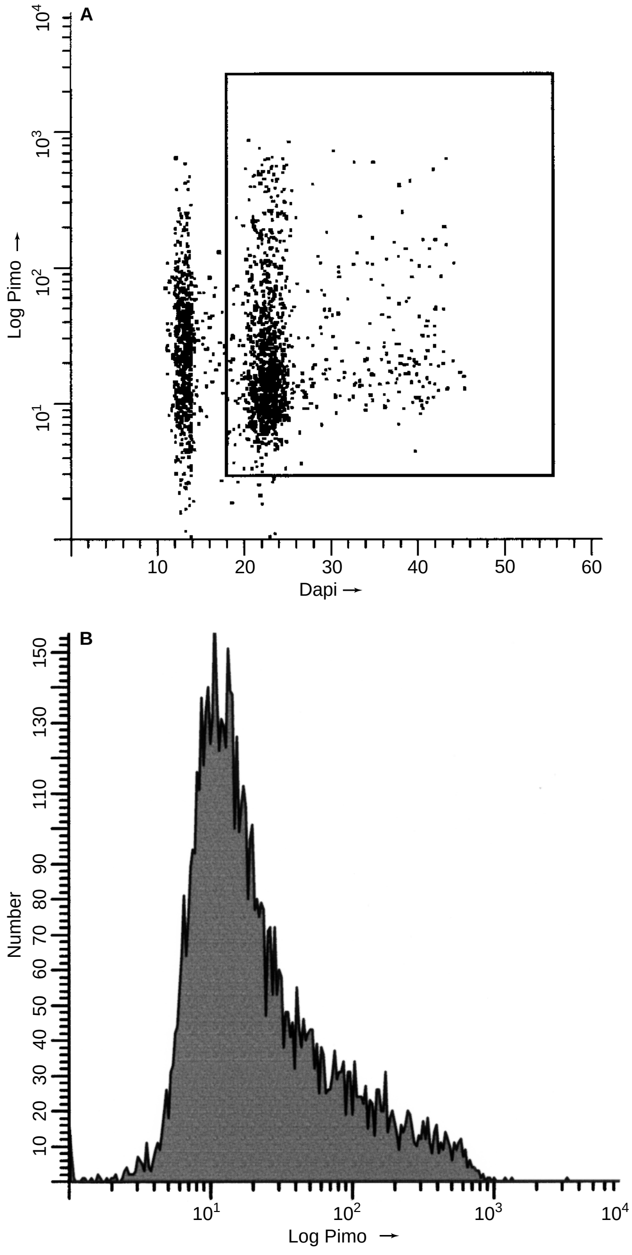

**Figure 1** Analysis of pimonidazole binding in an SCCVII tumour. A C3H mouse bearing a 400 mg SCCVII squamous cell carcinoma was injected intraperitoneally with 100 mg $kg^{-1}$ pimonidazole. 90 minutes later, the tumour was excised, and a single cell suspension was prepared. Single cells were fixed in 70% alcohol, then incubated with FITC-conjugated monoclonal antibody against pimonidazole, and DNA was stained with DAPI. Panel (**A**) shows the bivariate plot of DNA content versus pimonidazole binding. The square delineates the tetraploid tumour cell population that is shown in the histogram in panel (**B**)

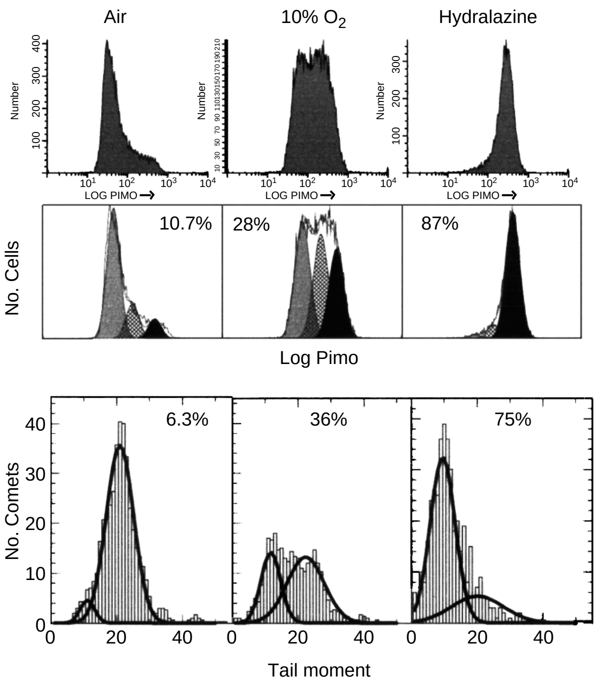

**Figure 2** Representative data from 3 SCCVII tumours analysed for pimonidazole binding and DNA damage. The upper curves show the distributions of anti-pimonidazole antibody binding in tumours from a mouse breathing air, 10% oxygen in nitrogen, or treated with hydralazine to reduce tumour blood flow. A fitting program was used to describe 3 normally distributed populations representing aerobic, intermediate and hypoxic tumour cells. The percentage of hypoxic cells is shown in the middle panels. For the same tumours, cells were analysed for hypoxic fraction using the comet assay that detects the difference in radiation-induced strand breaks between aerobic and radiobiologically hypoxic tumour cells. The percentage of hypoxic cells is given in each panel confined to the tetraploid tumour cell population, a procedure that was also adopted for comet analysis. However, it is apparent from the continuum of pimonidazole intensities seen in Figure 1 that simply setting a 'threshold' intensity to measure hypoxic fraction is highly subjective.

At least a 100-fold range in fluorescence intensity was observed for pimonidazole binding in SCCVII tumours (Figures 1, 2). In air-breathing mice, most of the tumour cells were low in fluorescence intensity, however, for mice breathing 10% oxygen during exposure to pimonidazole, there was a shift in average intensity to much higher values (Figure 2, upper panels). Most of the cells were brightly fluorescent when mice were injected intraperitoneally (i.p.) with hydralazine 15 min after i.p. injection of pimonidazole. Although hydralazine and clamping of the tumour will trap drug in the tumour and reduce subsequent access to the drug from plasma, there was little change in the average fluorescence of the hypoxic cells. The middle row in Figure 2 illustrates our objective curve-fitting procedure, where we used an unconstricted best fit of three Gaussian distributions representing aerobic, intermediate and hypoxic populations. The mean fluorescence of the aerobic distribution was on average 10.8 ± 0.95 (mean ± SE, $n$ = 43) times lower than the mean of the hypoxic distribution, although there was a trend for this ratio to increase for low hypoxic fractions and decrease for high hypoxic fractions. Sufficient differences in staining intensity between aerobic and hypoxic cells to justify the use of the fitting procedure were typically seen for pimonidazole concentrations exceeding 30 mg $kg^{-1}$.

© 2000 Cancer Research Campaign

For each tumour cell suspension, the comet assay was also used to measure the fraction of radiobiologically hypoxic cells (Figure 2, lower panels). For this measurement of hypoxia, tumours were exposed to radiation 90 min after injection of pimonidazole. This length of time was found to be adequate to reduce parent drug levels and thereby minimize any DNA breaks in hypoxic cells caused by pimonidazole radiosensitization. In control experiments, no DNA breaks by these tracer levels of pimonidazole were observed. Agreement between the two methods for quantifying the hypoxic population was best when the population with intermediate levels of antibody staining was not included in the pimonidazole hypoxic fraction.

To examine the reproducibility and sensitivity of the pimonidazole curve-fitting method for detecting hypoxic cells, aerobic or anoxic cells were incubated for 90 minutes with 20 μg $ml^{-1}$ pimonidazole, and then mixed together in given proportions, fixed in ethanol, and analysed for pimonidazole adducts (Figure 3). Anoxic cells were identified to a detection limit of 0.5% when 20 000 cells were analysed. Qualitatively similar results were obtained when cells were incubated with a 4-fold lower concentration of pimonidazole, but the detection limit decreased to 2%. The variability between samples is indicated by the plotted standard deviation for 4 measurements obtained from the same fixed cell mixture separated into individual samples for antibody binding and analysis. When analysis was delayed until 10 days after either fixation or even antibody labelling, the proportion of hypoxic cells was not significantly different (data not shown). Therefore reproducibility appears to be excellent and is unaffected by holding samples for several days after preparation.

Although it was not possible to verify directly that the cells that bound pimonidazole were the hypoxic cells in the tumour, the location of the pimonidazole positive cells relative to the blood supply could be examined with the expectation that cells distant from the functional blood vessels were likely to be hypoxic. Tumour cell subpopulations with different hypoxic fractions were sorted on the basis of a Hoechst 33342 fluorescence gradient produced after injection of this DNA stain into the tail vein of the mouse. As previously observed for tumours labelled in air-breathing mice (Durand and Raleigh, 1998), cells containing the highest concentrations of Hoechst 33342 (i.e., closest to blood vessels) showed the least amount of pimonidazole binding, and cells with the lowest concentrations of Hoechst (i.e., distant from the blood supply) bound the most pimonidazole (Figure 4).

A comparison between hypoxic fraction measured using the comet assay and hypoxic fraction measured using pimonidazole marker binding in the same SCCVII tumours is shown in Figure 5. The two methods were highly correlated for hypoxia. Interestingly, carbogen breathing did not produce a significant decrease in tumour hypoxic fraction relative to air-breathing. This is likely to be explained by the long carbogen breathing period (90 min) necessary for the pimonidazole binding studies. Although carbogen may initially reduce tumour hypoxic fraction, an extended breathing period (i.e., more than about 15 min) may counteract this effect for some tumour types (Falk et al, 1992; Hill et al, 1998).

Since pimonidazole binding is cell line dependent (Durand and Raleigh, 1998), the applicability of the pimonidazole curve-fitting analysis was also examined in two human tumour xenografts known to bind less pimonidazole on average than SCCVII. SiHa cervical carcinoma and U87 gliomas were used at tumour sizes containing about 20% and 9% hypoxic cells, respectively. Results for individual SCCVII tumours, as well as for the U87 and SiHa xenograft tumours are combined in Figure 6, indicating that the objective flow histogram analysis method is also applicable to human tumour xenografts.

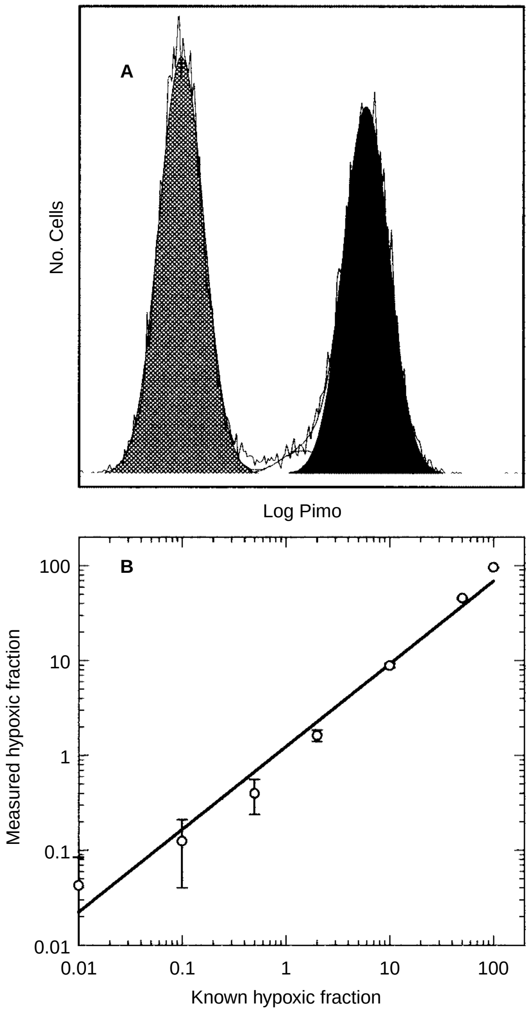

**Figure 3** Analysis of hypoxic cells in known mixtures using the flow cytometry method. Chinese hamster V79 cells were incubated in medium equilibrated with air or nitrogen containing 20 μg $ml^{-1}$ pimonidazole. After a 90 min incubation, cells were recovered, mixed in various proportions, and fixed in ethanol. Three days later, samples were analysed for pimonidazole adducts using flow cytometry. Panel (**A**) is a representative histogram for a mixture containing 46% hypoxic cells. Panel (**B**) shows the measured hypoxic fraction plotted versus the known hypoxic fraction for 4 samples per mixture (mean ± SD). The linear best-fit curve is shown, and the point on the y-axis shows the results for 0% hypoxic cells

In our first clinical evaluations, 3 patients with cervical carcinoma were given an intravenous infusion of 0.5 g $m^{-2}$ pimonidazole approximately 24 hours before tumour excision biopsy. Single cells obtained from these biopsies were subsequently analysed for hypoxic fraction using the objective flow cytometry method. Results shown in Figure 7 indicate hypoxic fractions ranging from 2.7% to 8.3%.

### DISCUSSION

Pimonidazole binding to hypoxic cells has been shown to be an effective way to identify hypoxic regions within solid tumours (Kennedy et al, 1997; Thrall et al, 1997; Durand and Raleigh,

© 2000 Cancer Research Campaign

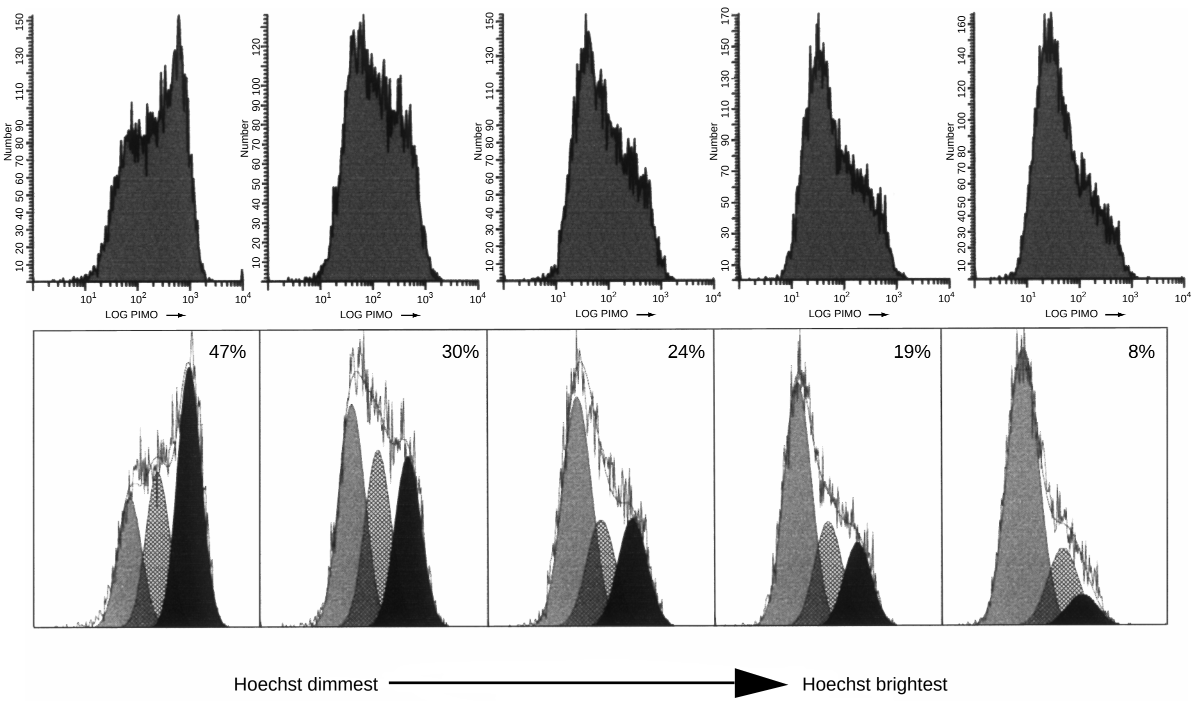

**Figure 4** Analysis of the response of tumour cells recovered from different regions of an SCCVII tumour from a mouse breathing 10% oxygen in nitrogen. The mouse was injected intraperitoneally with 100 mg kg$^{-1}$ pimonidazole, and 90 min later given an intravenous injection of 8 mg kg$^{-1}$ Hoechst 33342 to stain cells close to the vasculature. The single cell suspension prepared from this tumour was sorted on the basis of the Hoechst 33342 diffusion gradient into 5 equal fractions representing cells distant from the blood vessels (Hoechst dimmest) to cells closest to the blood vessels (Hoechst brightest). The percentage of cells calculated as hypoxic, based on the 3 population fit, is given in each panel

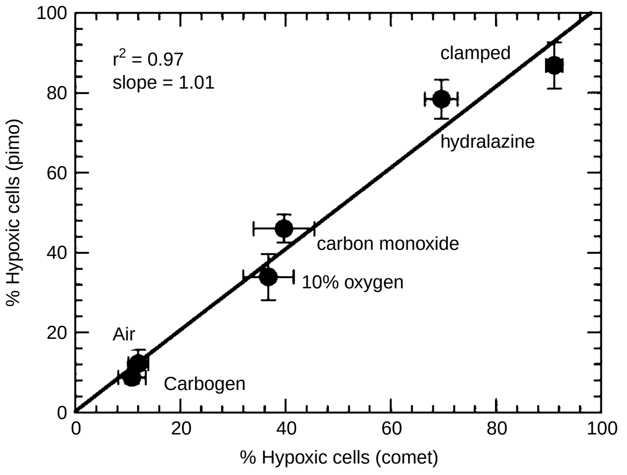

**Figure 5** Comparison between pimonidazole marker binding and comet assay for 45 SCCVII tumours. Tumours from mice breathing various gas mixtures, mice treated with hydralazine, or tumours that were clamped 20 min after pimonidazole injection, were compared for hypoxic fraction measured using pimonidazole binding, or by comet assay. The means and standard errors are shown

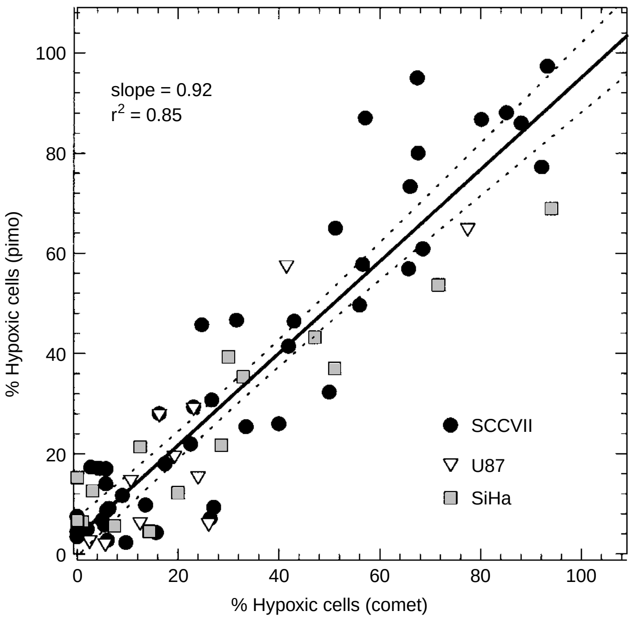

**Figure 6** Comparison between pimonidazole marker binding and comet assay for SCCVII tumours, U87 glioma xenograftsand SiHa cervical carcinoma xenografts. Results for individual tumours are shown, and the slope and correlation coefficient for the linear best-fit for all of the tumours are shown

1998; Varia et al, 1998; Raleigh et al, 1999). However, the degree of hypoxia associated with the pimonidazole labelled areas has not been well defined. For example, in the C3H mouse mammary carcinoma, a comparison was made between pimonidazole binding and two measures of tumour hypoxia: oxygen microelectrode measurements, and radiobiological hypoxic fraction as measured using a clamped tumour control endpoint (Raleigh et al, 1999). For both of these comparisons, only 30% of the tumour area was considered positive for pimonidazole binding, in spite of the fact that 100% of the tumour cells were radiobiologically

© 2000 Cancer Research Campaign

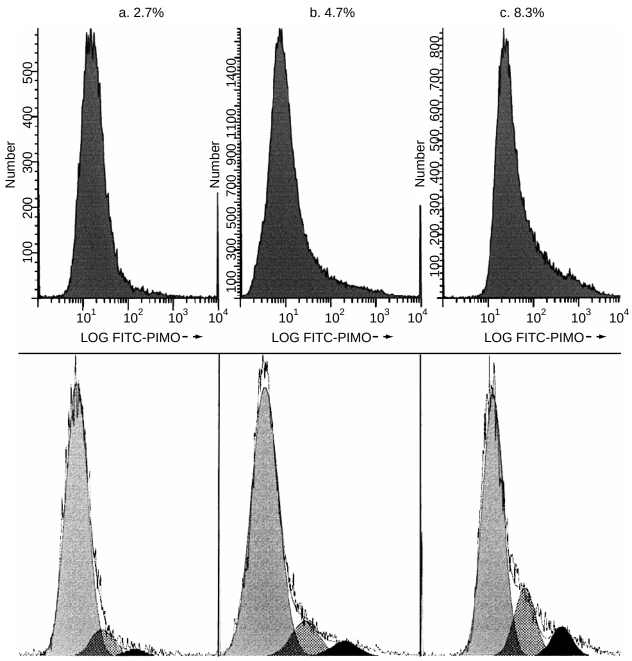

**Figure 7** Flow cytometry analysis of hypoxic fraction in cervical cancer cells from patients given 0.5 g $m^{-2}$ pimonidazole 24 hours prior to excision biopsy. The percentage of hypoxic cells derived from the curve-fitting programme is shown hypoxic or 100% of oxygen electrode measurements fell below 10 mmHg. Possible reasons for these underestimates have been discussed (Raleigh et al, 1999) and include lack of correction for necrotic regions in tumour sections, possible inability of pimonidazole to detect cells intermediate in oxygenation that still contribute to radiation resistance, or variable influence of intermittent changes in tumour perfusion on these different methods. In the present study, a direct comparison between the response of the single cells recovered from these tumours has shown a much stronger correlation between radiobiologic hypoxia and pimonidazole binding. In flow analysis, necrotic cells and normal diploid cells can be omitted from analysis, which should improve the correlation. An important advantage of this method over the comet assay is the ability to analyse tens of thousands of cells very rapidly, and thus identify small hypoxic fractions with more certainty. In attempting to estimate the sensitivity of the method for detecting hypoxic cells (Figure 3), an important issue is the number of cells analysed. Resolution will increase as the number of cells analysed increases. Rather than attempting to use absolute intensity for quantitation, flow analysis can make use of range in fluorescence intensity, or the percentage of cells above a background level of staining. This should avoid problems associated with variable drug delivery and differences in rate of drug metabolism.

A perceived problem with oxygen electrode measurements is that the relation between tumour $pO_2$ and radiobiologic hypoxia can vary for different murine tumour types (Horsman et al, 1994). Similarly, differences in rate of metabolism of pimonidazole between tumour types can lead to differences in rate of pimonidazole binding (Durand and Raleigh, 1998) and although clamping tumours can trap pimonidazole, it also reduces availability and causes less total pimonidazole to be bound. An important advantage of single cell analysis is that it provides information on the range of binding, and as indicated above, this range is what is critical for determining hypoxic fraction. Even though individual SiHa, SCCVII and U87 glioma tumours bound different absolute amounts of pimonidazole, the hypoxic fraction determined using the histogram-fitting method was found to be a useful measure of radiobiological hypoxic that was independent of differences in rate of pimonidazole binding between these tumour types. These results provide confidence that the flow cytometry method for evaluating hypoxia marker binding would also be applicable to cells from human tumours. A comparison was previously performed between the comet assay and binding of another hypoxia marker, EF5 (Kavanagh et al, 1999). These authors used an arbitrary factor of 10 times the background mean fluorescence as a cut-off to define the hypoxic cells of the tumour, and although the correlation between the comet assay and EF5 binding was very good, a comparison between both methods using the same cell suspension was not provided.

There are several ways to assess the sensitivity of the method for detecting hypoxic cells. From the slope and confidence limits shown in Figure 5, a value of about 5% hypoxia appears to represent the limit of sensitivity. However, errors associated with both pimonidazole staining and comet assay are included in this analysis, and the errors associated with the comet assay are larger than those associated with pimonidazole binding, at least for the lower hypoxic fractions (Figure 4). A more direct way of determining sensitivity of the pimonidazole binding method is by analysing flow histograms from known mixtures of aerobic and hypoxic cells. This analysis indicated that it could be possible to detect hypoxic fractions as low as 0.5%. However, mixtures of cultured cells used in Figure 3 represent an optimum situation that is unlikely to occur with heterogeneous samples obtained from human tumours. While the reproducibility of the method is excellent for multiple samples analysed from the same population, a larger error is associated with the curve-fitting programme itself. A comparison was made between results obtained independently by two investigators analysing 10 histograms from tumours that contained 3% to 95% hypoxic cells. While both investigators agreed on the percentage of aerobic cells in the samples ($r^2 = 0.99$, slope = 0.87), the percentage of cells defined as hypoxic or intermediate was more variable when hypoxic fractions exceeded 0.2. Modifications to the analysis programme to ensure similar CVs for the aerobic and hypoxic populations, and to maintain a 10-fold differential between the mean fluorescence of the aerobic and hypoxic populations should reduce this variability.

The 3 examples of pimonidazole-binding patterns in human tumours indicated a mean percentage of hypoxic cells of 5.3%. This value compares well with previous analysis of pimonidazole binding in tumour sections; the percentage of tumour sections from 13 cervical tumours that were labelled with pimonidazole averaged 4.3%, with a range of 0–25.8% (Raleigh et al, 1998). This average hypoxic fraction measured by pimonidazole binding can be compared to a mean oxygenation of about 10 mmHg for cervical tumours (Fyles et al, 1998), or a mean hypoxic fraction of 0.15 for a variety of tumour types analysed after radiation exposure using the comet assay (Olive et al, 1999).

In conclusion, comparison of results obtained using the comet assay with pimonidazole binding for the same tumour samples has provided an aid to interpret pimonidazole marker binding. The criteria used to define hypoxic cells by pimonidazole binding in a murine tumour were also applicable to two human tumour xenografts. Results obtained from cervical tumours from 3

 *© 2000 Cancer Research Campaign*

patients administered pimonidazole confirm that the method can be applied to single cells from excision biopsies, and an estimate of hypoxic fraction can be available on the day of biopsy. We are currently comparing this flow cytometry method with analysis of tumour sections from the same patient and with oxygen microelectrode measurements.

### ACKNOWLEDGEMENTS

This work was supported by USPHS grants CA-37879 and CA-50995 (to JAR), and by the National Cancer Institute of Canada with funds provided by the Canadian Cancer Society.

### REFERENCES

Aquino-Parsons C, Luo C, Vikse CM and Olive PL (1999) Comparison between the comet assay and the oxygen microelectrode for measurement of tumour hypoxia. *Radiother Oncol* **51**: 179–185

Brown JM and Giaccia AJ (1998) The unique physiology of solid tumors: opportunities (and problems) for cancer therapy. *Cancer Res* **58**: 1408–1416

Bush RS, Jenkin RDT, Allt WEC, Beale FA, Bean H, Dembo AJ and Pringle JF (1978) Definitive evidence for hypoxic cells influencing cure in cancer therapy. *Br J Cancer* **37**: 302–306

Chapman JD, Dugle DL, Reuvers AP, Meeker BE and Borsa J (1974) Studies on the radiosensitizing effect of oxygen in Chinese hamster cells. *Int J Radiat Biol* **26**: 383–389

Durand RE and Raleigh JA (1998) Identification of nonproliferating but viable hypoxic tumor cells *in vivo*. *Cancer Res* **58**: 3547–3550

Falk SJ, Ward R and Bleehen NM (1992) The influence of carbogen breathing on tumour tissue oxygenation in man evaluated by computerised p02 histography. *Br J Cancer* **66**: 919–924

Fyles AW, Milosevic M, Wong R, Kavanagh MC, Pintilie M, Sun A, Chapman W, Levin W, Manchul L, Keane TJ and Hill RP (1998) Oxygenation predicts radiation response and survival in patients with cervix cancer [published erratum appears in Radiother Oncol 1999 Mar; 50(3): 371]. *Radiother Oncol* **48**: 149–156

Hill SA, Collingridge DR, Vojnovic B and Chaplin DJ (1998) Tumour radiosensitization by high-oxygen-content gases: influence of the carbon dioxide content of the inspired gas on $PO_2$, microcirculatory function and radiosensitivity. *Int J Radiat Oncol Biol Phys* **40**: 943–951

Hockel M, Schlenger K, Aral B, Mitze M, Schaffer U and Vaupel P (1996) Association between tumor hypoxia and malignant progression in advanced cancer of the uterine cervix. *Cancer Res* **56**: 4509–4515

Horsman MR, Khalil AA, Siemann DW, Grau C, Hill SA, Lynch EM, Chaplin DJ and Overgaard J (1994) Relationship between radiobiological hypoxia in tumors and electrode measurements of tumor oxygenation. *Int J Radiat Oncol Biol Phys* **29**: 439–442

Kavanagh MC, Tsang V, Chow S, Koch C, Hedley D, Minkin S and Hill RP (1999) A comparison in individual murine tumors of techniques for measuring oxygen levels. *Int J Radiat Oncol Biol Phys* **44**: 1137–1146

Kennedy AS, Raleigh JA, Perez GM, Calkins DP, Thrall DE, Novotny DB and Varia MA (1997) Proliferation and hypoxia in human squamous cell carcinoma of the cervix: first report of combined immunohistochemical assays. *Int J Radiat Oncol Biol Phys* **37**: 897–905

Lee J, Siemann DW, Koch CJ and Lord EM (1996) Direct relationship between radiobiological hypoxia in tumors and monoclonal antibody detection of EF5 cellular adducts. *Int J Cancer* **67**: 372–378

McLaren DB, Pickles T, Thomson T and Olive PL (1997) Impact of nicotinamide on human tumour hypoxic fraction measured using the comet assay. *Radiother Oncol* **45**: 175–182

Olive PL (1989) Distribution, oxygenation, and clonogenicity of macrophages in a murine tumor. *Cancer Comm* **1**: 93–100

Olive PL (1995) Detection of hypoxia by measurement of DNA damage in individual cells from spheroids and murine tumours exposed to bioreductive drugs. I. Tirapazamine. *Br J Cancer* **71**: 529–536

Olive PL and Durand RE (1992) Detection of hypoxic cells in a murine tumor with the use of the comet assay. *J Natl Cancer Inst* **84**: 707–711

Olive PL, Banáth JP and Durand RE (1990) Heterogeneity in radiation-induced DNA damage and repair in tumor and normal cells measured using the "comet" assay. *Radiat Res* **122**: 86–94

Olive PL, Durand RE, LeRiche JC, Olivotto IA and Jackson SM (1993) Gel electrophoresis of individual cells to quantify hypoxic fraction in human breast cancers. *Cancer Res* **53**: 733–736

Olive PL, Horsman MR, Grau C and Overgaard J (1997) Detection of hypoxic cells in a C3H mouse mammary carcinoma using the comet assay. *Br J Cancer* **76**: 694–699

Olive PL, Durand RE, Jackson SM, LeRiche JC, Luo C, Ma R, McLaren DB, Aquino-Parsons C, Thomson TA and Trotter T (1999) The comet assay in clinical practice. *Acta Oncol* **38**: 839–844

Overgaard J (1994) Clinical evaluation of nitroimidazoles as modifiers of hypoxia in solid tumors. *Oncol Res* **6**: 509–518

Raleigh JA, Calkins-Adams DP, Rinker LH, Ballenger CA, Weissler MC, Fowler WCJ, Novotny DB and Varia MA (1998) Hypoxia and vascular endothelial growth factor expression in human squamous cell carcinomas using pimonidazole as a hypoxia marker. *Cancer Res* **58**: 3765–3768

Raleigh JA, Chou SC, Arteel GE and Horsman MR (1999) Comparisons among pimonidazole binding, oxygen electrode measurements, and radiation response in C3H mouse tumours. *Radiat Res* **151**: 580–589

Thrall DE, Rosner GL, Azuma C, McEntee MC and Raleigh JA (1997) Hypoxia marker labeling in tumor biopsies: quantification of labeling variation and criteria for biopsy sectioning. *Radiother Oncol* **44**: 171–176

Varia MA, Calkins-Adams DP, Rinker LH, Kennedy AS, Novotny DB, Fowler WCJ and Raleigh JA (1998) Pimonidazole: a novel hypoxia marker for complementary study of tumor hypoxia and cell proliferation in cervical carcinoma. *Gynecol Oncol* **71**: 270–277

Walton MI, Bleehen NM and Workman P (1989) Effects of localised tumour hyperthermia on pimonidazole (Ro 03–8799) pharmacokinetics in mice. *Br J Cancer* **59**: 667–673

Zhang H, Koch CJ, Wallen CA and Wheeler KT (1995) Radiation-induced DNA damage in tumors and normal tissues. III. Oxygen dependence of the formation of strand breaks and DNA-protein crosslinks. *Radiat Res* **142**: 163–168

© 2000 Cancer Research Campaign
